# Teacher noticing in K–12 science education: a literature review of its role in students’ scientific thinking

**DOI:** 10.3389/fpsyg.2026.1949423

**Published:** 2026-10-30

**Authors:** Hongxia Liu, Hongyang Zhang

**Affiliations:** 1 School of Teacher Development, Shaanxi Normal University, Xi’an, China; 2 Xi’an High-Tech Zone No. 3 Junior High School, Xi’an, China; 3 College of Physics and Information Technology Science, Shaanxi Normal University, Xi’an, China

**Keywords:** K–12 science education, professional vision, scientific sensemaking, scientific thinking, teacher noticing

## Abstract

Teacher noticing refers to how educators attend to, interpret, and respond to evidence of students’ thinking during classroom interactions. This integrative narrative review examined how teacher noticing may shape opportunities for scientific thinking in K–12 science education. Scientific thinking was conceptualized as inquiry, systematic observation, evidence evaluation, explanation construction, conceptual revision, and collaborative knowledge building. This review searched Web of Science, Scopus, Google Scholar, and relevant reference lists, and documented the study identification and selection process using a flow diagram adapted from PRISMA 2020. These procedures identified 17 empirical studies for integrative thematic synthesis. The synthesis suggests that teacher noticing involves selective attention, interpretation of students’ scientific ideas, and instructional response. Across the reviewed studies, teachers attended to students’ everyday language, tentative explanations, questions, drawings, classroom artifacts, inquiry practices, and participation. They interpreted the disciplinary and epistemic significance of these expressions and, in some cases, formulated or enacted responsive instructional moves. However, the evidence was strongest for teachers’ attention to and interpretation of student thinking. Enacted instructional responses were examined less frequently, and direct evidence linking teacher noticing to sustained student-level development remains limited. The findings therefore support a recursive account of noticing rather than a fixed sequence. Instructional responses may generate new student expressions that become available for further attention and interpretation. Overall, teacher noticing is best understood as a knowledge-guided, discipline-specific, and developable professional practice. It may shape the instructional and epistemic conditions for scientific reasoning, sensemaking, and knowledge construction. Future research should connect classroom-embedded measures of teacher noticing with longitudinal evidence of students’ reasoning, evidence use, explanation, and conceptual development.

## 1 Introduction

Cultivating scientific thinking involves more than the passive acquisition of established scientific content knowledge. It requires students to engage actively in dynamic cognitive processes. These processes include formulating research questions, testing hypotheses, and designing investigations. They also include evaluating data, constructing causal explanations, and revising mental models (Díaz et al., 2023; García-Carmona, 2025; Kuhn, 1993; Zimmerman, 2007). Contemporary science education research has therefore moved beyond assessing students solely through their mastery of established scientific knowledge. It increasingly examines students’ active participation in classroom knowledge construction. This shift is evident in frameworks focused on epistemic practices, which emphasize how students construct, evaluate, and revise knowledge through participation in disciplinary activity (Sezen-Barrie et al., 2020; Kang, 2024).

However, identifying scientific thinking in authentic classroom contexts is challenging for teachers. Student reasoning is often informal, fragmented, and context dependent. These features make it difficult to observe directly or assess fully through traditional instruments. One way to characterize scientific thinking more concretely is through students’ participation in interrelated scientific practices, as emphasized by the National Research Council (2012). These practices include developing and using models, analyzing and interpreting data, constructing explanations, and engaging in argument from evidence. Similarly, Windschitl et al. (2008) argued that traditional enactments of the scientific method emphasized testing predictions rather than ideas, whereas model-based inquiry engages students in generating, testing, and revising models. Students’ participation in these practices can make aspects of their scientific reasoning more visible in classroom interactions, enabling teachers to interpret that reasoning, make responsive instructional decisions, and support scientific sensemaking.

Teacher noticing refers to teachers’ ability to attend to important classroom events. It also involves interpreting what these events reveal about students’ thinking and evaluating their instructional significance. These processes are particularly important because classroom events often provide evidence of students’ thinking. The construct is conceptually linked to the broader sociological and cognitive framework of “professional vision”. Professional vision describes how professionals learn to notice and interpret complex events in specialized activity settings (Goodwin, 1994; Sherin and van Es, 2009). Research on video clubs has shown that video-based professional development can strengthen teachers’ professional vision. Video clubs provided structured opportunities for teachers to watch and discuss classroom video excerpts. Through these discussions, teachers refined both what they noticed and how they interpreted classroom interactions (Sherin and van Es, 2009). In science classrooms, productive disciplinary engagement depends on supportive classroom conditions. Teachers may help create these conditions by supporting students’ intellectual participation. Students can problematize content, exercise authority, follow discipline-specific expectations, and use meaningful resources for learning (Engle and Conant, 2002). In addition, attending to students’ epistemological framing can help teachers develop a more nuanced understanding of classroom engagement. This framing concerns how students understand their own activity in relation to knowledge and learning. This perspective helps teachers reinterpret seemingly unproductive classroom behaviour. Such behaviour may reflect students’ meaningful attempts to make sense of scientific phenomena (Hutchison and Hammer, 2010). Taken together, these perspectives suggest that teacher noticing is not merely perceptual. It is an interpretive practice that requires careful attention to the epistemic significance of students’ contributions.

Despite these conceptual insights, the link between teacher noticing and students’ scientific thinking requires further empirical explanation. Previous research in K–12 science classrooms has often examined these two areas separately. One strand of research has focused on teacher noticing and professional learning. This work has examined how teacher candidates and practicing teachers noticed, analyzed, and reflected on student thinking. These studies have examined these processes in practice rehearsals, classroom enactments, and video-based retrospective analyses (Arias et al., 2024; Barnhart et al., 2025; Luna and Sherin, 2017). Another strand of research has examined students’ engagement in inquiry, laboratory investigations, scientific sensemaking, and reasoning. These studies have shown how learners connected hands-on activity with minds-on reasoning. They also showed how learners constructed causal explanations and participated in collaborative knowledge-building discourse (Krist et al., 2023; Spaan et al., 2025; Talanquer et al., 2013; Tang et al., 2010). However, the relationship between teacher noticing and students’ scientific thinking remains underexamined. Existing research provides limited evidence tracing how teachers use noticing to guide instructional decisions and how those decisions may sustain and deepen students’ ongoing scientific thinking. This gap matters because scientific thinking is not merely the acquisition of canonical knowledge. Rather, scientific thinking is an iterative and nonlinear process of questioning, reasoning, explanation building, and model revision (Kuhn, 1993, 2002).

Effective teacher noticing extends beyond passive monitoring of classroom activity. Teachers need to interpret the disciplinary and epistemic value of students’ ideas. They also need to translate these interpretations into responsive pedagogical actions. These actions may include pressing for deeper mechanistic reasoning, connecting student ideas, and providing adaptive instructional support. Without this integration, science instruction may remain centered on factual recall or procedural completion. Such instruction may not support authentic scientific reasoning, sensemaking, and collective knowledge construction. This review synthesizes empirical research on teacher noticing in K–12 science education. It examines how teacher noticing relates to the development of students’ scientific thinking in classroom contexts.

In China, the revised Compulsory Education Physics Curriculum Standards explicitly identify scientific thinking as a core competency in school physics education (Ministry of Education of the People's Republic of China, 2022). The focus on K–12 science education is intentional. This review examines how teachers notice and respond to students’ emerging scientific thinking within school-based science instruction, where students often express their ideas through everyday language, tentative explanations, representations, and participation in inquiry activities. Focusing on K–12 contexts provides a defined educational scope for examining how teachers recognize and interpret these expressions and use them to inform instructional decisions. Although teacher noticing is also relevant to higher education, differences in learning contexts, curricular expectations, and student populations warrant consideration beyond the scope of the present review. Studies involving preservice teachers were included when their noticing activities concerned K–12 science teaching rather than university-level science learning.

In this review, scientific thinking is not conceptualized as a discrete cognitive skill. Instead, it is viewed as a dynamic form of participation in the scientific practices. Students express scientific thinking when they generate questions, make systematic observations, and evaluate empirical evidence. They also express scientific thinking when they construct and revise explanations, engage in sensemaking, and exercise epistemic agency during knowledge construction (National Research Council, 2012; NGSS Lead States, 2013). These forms of participation align with the concept of productive disciplinary engagement proposed by Engle and Conant (2002). In this framework, learners address disciplinary problems and use evidence to develop arguments. Learners also remain accountable to disciplinary norms and shared intellectual standards. Accordingly, the present synthesis treats these forms of participation as observable forms of students’ scientific thinking in classrooms. The analysis focuses on how teachers attend to these forms of reasoning. It also examines how teachers interpret the disciplinary and epistemic significance of these manifestations. Finally, the synthesis considers how teachers design instructional responses that support coherent inquiry, evidence-based argumentation, and knowledge construction. To clarify how teacher noticing has been conceptualized and examined in K–12 science education, and to evaluate the available evidence concerning its relationship with students’ scientific thinking, this review addresses the following two research questions:

1 How has teacher noticing been conceptualized and operationalized within the existing literature?
2 Through what pathways does teacher noticing support students’ scientific thinking in K–12 classrooms?

By addressing these questions, this review makes three contributions with both theoretical and practical implications. First, it clarifies the conceptual relationship between teacher noticing and students’ scientific thinking. It shows how students’ reasoning becomes observable through their context-specific ideas, participation in inquiry practices, evidence use, explanations, sensemaking, and epistemic agency. Second, it integrates findings from 17 empirical studies. These studies illustrate how teachers’ attention to, interpretation of, and instructional responses to students’ reasoning may support diverse forms of scientific thinking. Third, it identifies implications for science teacher education and professional development. It emphasizes that teachers need support not only to identify students’ ideas but also to interpret their disciplinary and epistemic significance. It also highlights how teachers may use questioning, feedback, and task design to sustain inquiry and collaborative knowledge construction. The remainder of this article describes the review method, synthesizes the findings, discusses implications, and outlines directions for future research.

## 2 Scientific thinking

### 2.1 Definition and theoretical basis of scientific thinking

Scientific thinking has been widely discussed in science education, yet no universally accepted definition has been established. The term has been defined and used in different ways (Díaz et al., 2023; García-Carmona, 2025). Kuhn (1993) argued against treating scientific thinking as a rarefied form of thought. Instead, Kuhn situated it within “the realm of the ordinary” (p. 319). Kuhn also emphasized that scientific thinking does not “come naturally” (p. 321). Accordingly, the development of scientific thinking requires deliberate practice, structured support, and targeted instruction. Kuhn (2002) later described scientific thinking as intentional “knowledge seeking” (p. 498). This description highlights its active, dynamic, and process-oriented nature. Zimmerman (2007) expanded this view by describing scientific thinking as involving “thinking and reasoning skills” (p. 172). These skills include “inquiry, experimentation, evidence evaluation, and inference” (p. 172). Klahr and Dunbar (1988) further conceptualized scientific reasoning as a dynamic and iterative process in which hypothesis generation, experimental design, and evidence evaluation interact. In their dual-space model, scientific reasoning involves search within a hypothesis space and an experiment space. Experimental outcomes may contribute to the development of new knowledge, which in turn gives rise to new hypotheses and different strategies of investigation and analysis. In this review, scientific thinking is treated as the broader construct, whereas scientific reasoning refers more specifically to the cognitive processes through which students generate and test hypotheses, evaluate evidence, draw inferences, and construct or revise explanations and models. Scientific reasoning is therefore regarded as a central component of scientific thinking.

### 2.2 Scientific thinking as classroom practice

Although scientific thinking involves internal cognitive processes, aspects of it become visible through students’ participation in classroom practices and interactions. In science learning environments, students demonstrate scientific thinking when they pose questions, make systematic observations, evaluate evidence, construct causal explanations, develop and revise models, and engage in collaborative sensemaking. Because these emerging forms of thinking and reasoning are often partial, informal, and context dependent, they cannot be adequately understood by evaluating final correct answers alone. Morris et al. (2013) argued that scientific thinking skills “do not routinely develop” and “must be scaffolded via educational and cultural tools” (p. 1). They also described scientific thinking as “highly educationally mediated” and dependent on “the cultural tools of science” (p. 2). These tools include “language”, “technology”, “computer simulations”, and “meta-cognitive scaffolding” (p. 3). From this sociocultural perspective, the development of scientific thinking is supported by carefully designed learning environments and discursive spaces. Such spaces allow students to express, test, critique, and refine their ideas.

### 2.3 Scientific thinking and teacher noticing

The development of students’ scientific thinking depends on students’ cognition and engagement. It may also depend on teachers’ professional capacity to attend to, interpret, and respond to students’ thinking in real time. Foundational frameworks of teacher noticing, developed largely in mathematics education, conceptualize teacher noticing as involving three central processes. Teachers attend to significant classroom events, interpret students’ mathematical thinking, and make instructional decisions based on those interpretations (Jacobs et al., 2010; Van Es and Sherin, 2002). Similarly, Hoth et al. (2022) emphasized that mathematics teachers need to be “highly attentive to students’ thinking, learning difficulties, and any misconceptions” and should “analyze students’ thinking” to determine “how best to react” (p. 1). This view connects teacher noticing closely to teachers’ interpretation of and response to students’ mathematical thinking. Although these studies focus on mathematical thinking, their emphasis on attending, interpreting, and responding provides a useful conceptual basis for examining how science teachers notice students’ scientific thinking.

In science classrooms, this noticing capacity is especially important because students’ scientific thinking is often emergent. It may be expressed in nonstandard forms. These forms include everyday language, fragmented explanations, spontaneous questions, preliminary drawings, gestures, mechanistic hypotheses, and inquiry practices (Krist et al., 2023; Luna et al., 2018; Mercier and Hinman, 2025; Mercier et al., 2026; Stammes and de Putter-Smits, 2025). Consequently, teachers need professional vision that enables them to look beyond formally correct answers. They must also attend to students’ logic, use of evidence, explanatory structures, and sensemaking efforts as expressed in their talk and work. From this perspective, teacher noticing functions as a cognitive and pedagogical bridge. It connects students’ emerging reasoning with responsive and discipline-specific instruction.

## 3 Review approach

### 3.1 Search strategy and study selection

This article adopts an integrative narrative review approach. Erol (2022) noted that narrative and integrative reviews are forms of traditional reviews that have increasingly adopted more comprehensive and systematic approaches. Structured search, selection, and data-extraction procedures were used to enhance transparency and coverage. The review aimed to develop a critical conceptual and thematic integration rather than to estimate effects. The searches were conducted at two time points because the initial search yielded relatively few studies directly relevant to the research questions, prompting a refinement of the search terms and a subsequent search to improve the retrieval of relevant literature.

The study-identification and selection process is reported in a flow diagram adapted from PRISMA 2020 (Page et al., 2021), as shown in Figure 1. This diagram was used to enhance the transparency of study identification and selection, rather than to indicate that the present study was conducted as a systematic review. In April 2026, database searches were conducted in Scopus and Web of Science. The searches aimed to identify peer-reviewed empirical studies examining teacher noticing in relation to students’ scientific thinking in K–12 science education. In May 2026, the searches in Scopus and Web of Science were repeated using refined search terms, and additional searches were conducted in Google Scholar. The reference lists of the database-derived studies and relevant review articles were also examined. Web of Science and Scopus were chosen for their broad, multidisciplinary coverage, while Google Scholar was used to broaden the search. However, excluding databases specific to education, such as ERIC and Education Source, may have limited the comprehensiveness of this review.

**Figure 1:**
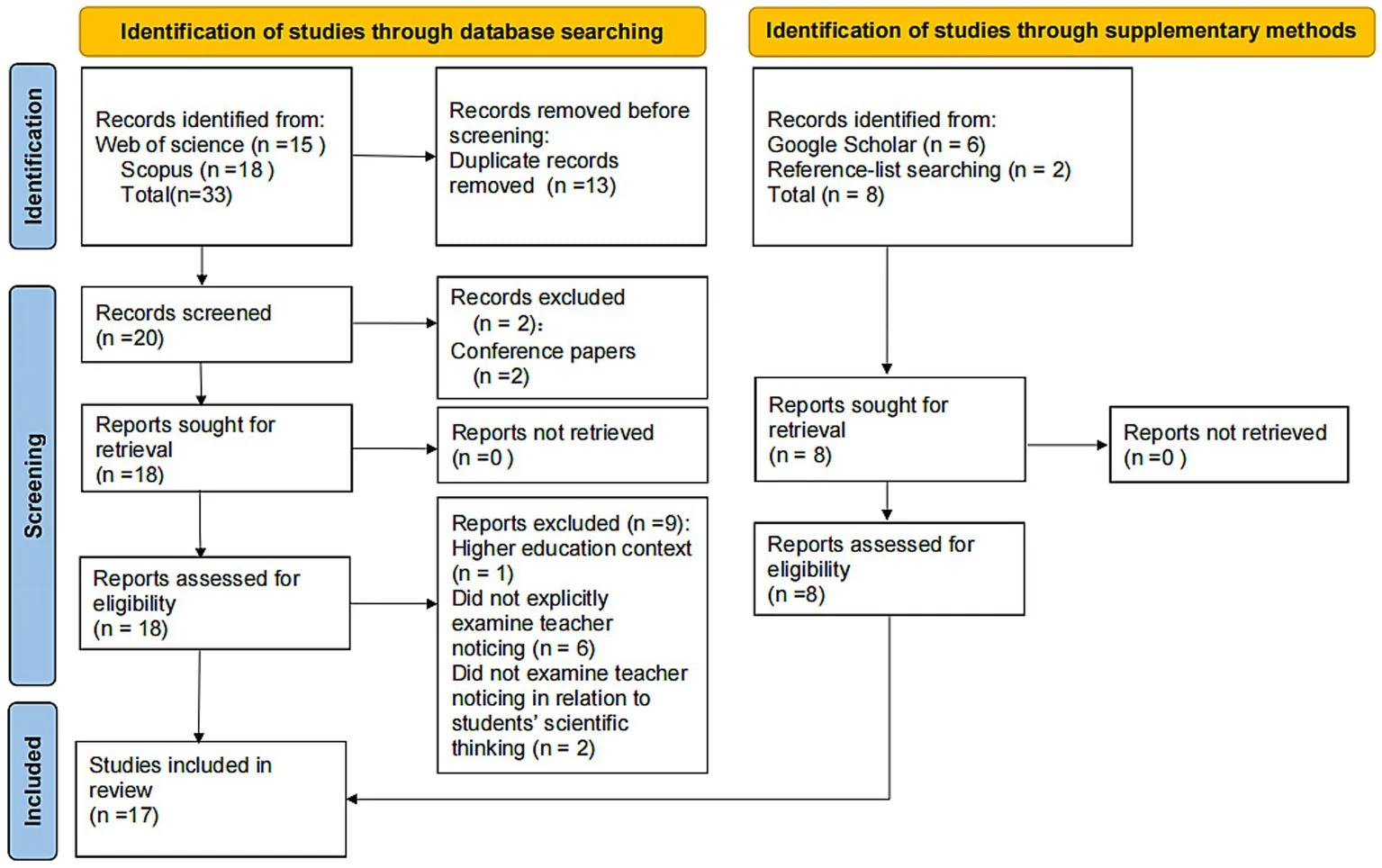
Flow diagram of study identification and selection, adapted from PRISMA 2020.

Search terms were informed by established theoretical, empirical, and review literature on teacher noticing and scientific thinking. All identified records were assessed against the predefined eligibility criteria.

To improve the relevance and coverage of the search, the teacher noticing terms were informed by prior reviews in science and mathematics education. Specifically, the search terms drew on the review by Chan et al. (2021), Teacher Noticing in Science Education: Do You See What I See?, as well as the review from Weston and Amador (2023), Teacher Noticing: A Literature Review of Mathematics and Science Teacher Noticing Conceptualizations. Based on these reviews, the search block included terms related to teacher noticing and closely related constructs, including “teacher noticing”, “professional noticing”, “professional vision”, “teacher attention”, and “teacher selective attention”.

The scientific thinking search terms were informed by conceptual and theoretical literature on scientific reasoning and science education. The review written by Díaz et al. (2023) informed the terms related to scientific thinking and reasoning. The theoretical analysis of García-Carmona (2025) further informed the distinction between scientific thinking and related intellectual processes. The search block covered scientific reasoning, inquiry, sensemaking, explanation, argumentation, scientific practices, and science process skills. The complete search strings are presented in Table 1.

**Table 1:** Database search strategies for teacher noticing and students’ scientific thinking.

	Search query and combinations	Number of search results
Database: web of science		
1	TS = (“teacher noticing” OR “teachers noticing” OR “teacher’s noticing” OR “teachers’ noticing” OR “professional noticing” OR “teacher professional noticing” OR “professional vision” OR “teacher attention” OR “teacher’s attention” OR “teachers’ attention” OR “teachers attention” OR “teacher selective attention” OR “teachers selective attention”)	1,025
2	TS = (“scientific thinking” OR “scientific reasoning” OR “scientific inquiry” OR “scientific sense making” OR “scientific explanation*” OR “scientific argumentation” OR “scientific practice*” OR “science process skill*”)	11,158
3	TS = (“science education” OR “science teaching” OR “science classroom*” OR “science lesson*” OR biology OR chemistry OR physics)	1,678,354
4	TS = (“k–12” OR k12 OR “elementary school*” OR “primary school*” OR “middle school*” OR “junior high school*” OR “senior high school*” OR “secondary school*” OR “high school*” OR pupil* OR student* OR learner* OR “school child*”)	1,070,865
5	1 AND 2 AND 3 AND 4	15
Database: scopus		
1	TITLE-ABS-KEY (“teacher noticing” OR “teachers noticing” OR “teacher’s noticing” OR “teachers’ noticing” OR “professional noticing” OR “teacher professional noticing” OR “professional vision” OR “teacher attention” OR “teacher’s attention” OR “teachers’ attention” OR “teachers attention” OR “teacher selective attention” OR “teachers selective attention”)	1,701
2	TITLE-ABS-KEY (“scientific thinking” OR “scientific reasoning” OR “scientific inquiry” OR “scientific sense making” OR “scientific explanation*” OR “scientific argumentation” OR “scientific practice*” OR “science process skill*”)	21,663
3	TITLE-ABS-KEY (“science education” OR “science teaching” OR “science classroom*” OR “science lesson*” OR biology OR chemistry OR physics)	5,307,909
4	TITLE-ABS-KEY (“k–12” OR k12 OR “elementary school*” OR “primary school*” OR “middle school*” OR “junior high school*” OR “senior high school*” OR “secondary school*” OR “high school*” OR pupil* OR student* OR learner* OR “school child*”)	2,826,650
5	1 AND 2 AND 3 AND 4	18

In Web of Science, searches were conducted using the Topic (TS) field. In Scopus, searches were conducted using the TITLE-ABS-KEY field. The complete search strategies and results are presented in Table 1. The refined database searches conducted in May 2026 returned 33 records: 15 from Web of Science and 18 from Scopus. All records were imported into Zotero. Automated and manual deduplication removed 13 records, leaving 20 unique records for title and abstract screening. The subsequent study-selection process is summarized in Figure 1.

### 3.2 Inclusion and exclusion criteria

A predefined set of inclusion and exclusion criteria was used to evaluate the eligibility of the retrieved records. Studies were included in the final synthesis if they met the following five criteria: (i) they were published in English; (ii) they appeared as peer-reviewed journal articles; (iii) they used empirical research designs, including qualitative, quantitative, or mixed-methods approaches; (iv) they were situated in K–12 science education contexts or directly involved preservice or in-service teachers working in K–12 science instruction; and (v) they explicitly examined teacher noticing, professional noticing, professional vision, teacher attention, or closely related instructional-attention processes in relation to students’ scientific thinking or related science-learning processes. The eligibility criteria were first applied to 20 unique records during title and abstract screening. Two conference papers were excluded, leaving 18 reports for full-text assessment. The full texts of all 18 reports were retrieved and assessed. Nine reports were excluded at this stage. One was situated in a higher education context, six did not explicitly examine teacher noticing or a closely related instructional-attention process, and two did not examine these processes in relation to students’ scientific thinking or related science-learning processes. The remaining nine database-derived studies were included in data extraction and integrative thematic synthesis.

A supplementary search was conducted in May 2026 to identify studies that may not have been captured by the database searches. Google Scholar was searched using combinations of the core terms, and the first 20 results from each search were screened. The reference lists of the nine database-derived studies and relevant review articles were also examined. All records were assessed using the same eligibility criteria. Eight additional studies were included, with six identified through Google Scholar and two through reference-list searching. Together with the nine database-derived studies, the final corpus comprised 17 empirical studies.

### 3.3 Data extraction and synthesis

Data were extracted and organized using a standardized Microsoft Excel literature grid. Each of the 17 included studies was read closely. Extracted information included authors, publication year, geographic location, research context, participant characteristics, research design, data collection methods, analytical approach, and key findings. Cross-study comparisons were then conducted to identify recurring conceptual patterns and empirical themes. Study selection, data extraction, and thematic coding were conducted solely by the first author, which represents a methodological limitation.

The descriptive synthesis mapped the geographic distribution, participant groups, methodological designs, and data sources of the included studies. The integrative thematic synthesis examined how teacher noticing was conceptualized and how it was linked to students’ scientific thinking. Supplementary Table S1 distinguishes between direct and indirect evidence of teacher noticing. Coding and theme development were conducted iteratively and were informed by established thematic synthesis and literature-grid approaches (Thomas and Harden, 2008; Yang and Whatman, 2025). No standardized quality or risk-of-bias appraisal was conducted. Although this review focuses on conceptual synthesis, the absence of a formal quality appraisal limits the assessment of the methodological quality of the included studies.

The synthesis was organized around the two review questions.

## 4 Findings

### 4.1 Research question 1: conceptualizing and operationalizing teacher noticing

Teacher noticing has become an important construct in research on teachers’ professional competence, classroom decision-making, and professional development. It has been used to examine how teachers selectively attend to classroom events, interpret them in relation to student thinking and broader principles of teaching and learning, and draw on professional and contextual knowledge to inform subsequent instructional decisions and responses (Barnhart and van Es, 2015; Jacobs et al., 2010; König et al., 2022; Van Es and Sherin, 2002). In science education, the objects of noticing have included students’ scientific ideas, inquiry practices, representations, reasoning, participation, and sensemaking. However, the included studies also showed that teachers did not consistently prioritize students’ scientific thinking and sometimes focused instead on their own teaching, student behaviour, answer correctness, or procedural aspects of inquiry (Sharma, 2025; Talanquer et al., 2013; Tang et al., 2010). Although terms such as teacher attention, professional vision, professional noticing, reflective noticing, and noticing students’ thinking have been used in partially overlapping ways (Chan et al., 2021; Weston and Amador, 2023; Weyers et al., 2023), this review uses teacher noticing as an umbrella term for teachers’ selective attention to, interpretation of, and instructional use of evidence of student thinking.

#### 4.1.1 Components and objects of teacher noticing

To interpret the included studies, this review also draws on foundational conceptual literature that was not part of the 17-study empirical corpus. These foundational accounts commonly conceptualize teacher noticing as involving selective attention, interpretation, and instructional response. Van Es and Sherin (2002) described teacher noticing as involving three related activities:

Teacher noticing involves (a) identifying what is important or noteworthy about a classroom situation; (b) making connections between the specifics of classroom interactions and the broader principles of teaching and learning they represent; and (c) using what one knows about the context to reason about classroom interactions. (p. 573).

Jacobs et al. (2010) offered a more specific definition of professional noticing in relation to children’s mathematical thinking, describing it as “a set of three interrelated skills: attending to children’s strategies, interpreting children’s understandings, and deciding how to respond on the basis of children’s understandings” (p. 172).

Although developed in different contexts, these definitions share a common structure. Teachers select particular classroom events or student contributions for attention, interpret what they may reveal about student thinking, and may use those interpretations to inform instructional decisions. Classroom events therefore do not constitute self-evident evidence; their significance depends on teachers’ professional knowledge, expectations, experience, and framing. König et al. (2022) similarly argued that “teachers’ expertise—and thus their noticing—develops over different stages through cognition and reflection related to teaching practices and experiences” (p. 2).

Across the included science education studies, the objects of noticing fell into three overlapping groups. First, teachers attended to the forms through which student thinking was expressed, including everyday language, tentative explanations, personal experiences, drawings, written work, and other classroom artifacts (Luna et al., 2018; Mercier and Hinman, 2025; Mercier et al., 2026; Stammes and de Putter-Smits, 2025). Second, teachers attended to the disciplinary and epistemic substance of student contributions, including scientific concepts, inquiry practices, evidence use, mechanistic reasoning, the origins and relationships of ideas, and orientations toward knowledge construction (Krist et al., 2023; Russ, 2018; Talanquer et al., 2013; Tang et al., 2010). Third, some studies examined participation, emotion, social interaction, epistemic agency, and changes in collective inquiry over time (Kozaklı Ülger et al., 2026; Krist et al., 2023; Park and Zhang, 2025). Teacher noticing in science education was therefore conceptualized through both general components—attention, interpretation, and response—and the science-specific forms of thinking, representation, and participation toward which these components were directed.

#### 4.1.2 Research contexts, data sources, and evidentiary reach

Across the included studies, teacher noticing was operationalized and examined through written reflections and performance assessments, video-based tasks, teaching rehearsals and post-rehearsal discussions, interviews, student-generated artifacts, classroom observations, point-of-view video, and longitudinal teacher journals (Table 2; Supplementary Table S1). These approaches yielded different forms of evidence concerning attention or recognition, interpretation, planned response, enacted response, and student-level processes.

**Table 2:** Extraction of descriptive information from the 17 included articles.

Number	Authors	Geographical location	Title	Journal	Research method	Research participants	Data collection method	Data analysis techniques	Main findings	Relevancy to research
Publication 1	Arias et al. (2024)	United States	Preservice elementary teachers’ noticing in reflections of rehearsal and classroom enactments during practice-based methods courses across three universities	Teaching and Teacher Education	Qualitative multiple-case study	Preservice elementary teachers from three universities	Written reflections and course-based artifacts	Deductive and inductive coding	PSTs showed holistic noticing of teacher–student interactions across rehearsal and classroom reflections. Evidence-based reasoning appeared more often in Explore rehearsals and classroom lessons.	High
Publication 2	Benedict-Chambers (2016)	United States	Using tools to promote novice teacher noticing of science teaching practices in post-rehearsal discussions	Teaching and Teacher Education	Qualitative study	16 novice teachers in an elementary science methods course	Post-rehearsal discussion videos and transcripts	Coding of discussion topics and discourse moves	Tool-supported post-rehearsal discussions helped novice teachers focus more on student learning, science concepts, and scientific practices rather than general pedagogy.	High
Publication 3	Benedict-Chambers and Aram (2017)	United States	Tools for teacher noticing: helping preservice teachers notice and analyze student thinking and scientific practice use	Journal of Science Teacher Education	Qualitative study	53 novice elementary teachers	Written reflections from three teaching rehearsals	Coding of reflection revisions and justifications; content analysis of noticing tools	Most revisions focused on students’ science content learning and scientific practices, while fewer addressed general pedagogy.	High
Publication 4	Mercier and Hinman (2025)	United States	Preservice teachers’ framing and noticing of elementary students’ ways of communicating scientific ideas	Journal of Research in Science Teaching	Exploratory qualitative case study	44 preservice teachers	Written explanations, noticing-session reflections, and group discussion recordings	Coding and noticing profile analysis	PSTs’ noticing shifted toward more equitable and anti-deficit interpretations, though this shift was uneven and non-linear.	High
Publication 5	Mercier et al. (2026)	United States	Designing for more equitable noticing: creating and refining protocols to support preservice teacher noticing	Journal of Science Teacher Education	Design-based research	87 preservice teachers	video cases; notebook entries; structured small-group discussions	coding and analytic memos	Structured noticing protocols helped PSTs develop more asset-based noticing of students’ scientific sensemaking.	High
Publication 6	Kang (2024)	South Korea	Reappraisal of doing physics: role of epistemic agency and cultural practices	New Physics: Sae Mulli	Literature review and empirical classroom video analysis	Five middle school science teachers (4 females, and 1 male)	Classroom video recordings, textbooks, and student worksheets	Video analysis; 80 episodes of students’ involvement in scientific practices identified and coded based on the eight NGSS scientific practices.	Several teaching strategies facilitated students’ sensemaking, while a missed opportunity illustrated the importance of noticing and responding to students’ emerging reasoning.	High
Publication 7	Spaan et al. (2025)	Netherlands	Thinking-back-and-forth in practical work experienced by students: identifying evidence-informed characteristics of good practices in secondary education	Research in Science & Technological Education	Mixed-methods observational study	11 physics teachers, four chemistry teachers, and 303 students across 15 Grade 8–9 practical lessons in 13 Dutch secondary schools.	Classroom video observations, student learner reports, and post-lesson teacher interviews.	Video analysis; learner reports analysed for minds-on remarks; multilevel binary logistic model and qualitative analysis	Substantial attention to TBF, congruent pedagogy, and mitigation of hands-on cognitive load were associated with more self-reported minds-on learning experiences.	High
Publication 8	Tang et al. (2010)	United States	The scientific method and scientific inquiry: tensions in teaching and learning	Science Education	Qualitative case study/discourse analysis	One ninth-grade environmental science class and its teacher in a United States public high school	Classroom video recordings, teacher interviews, and videotaped teacher meetings	Discourse analysis of classroom discourse and teacher discourse	Focusing on the scientific method as discrete steps can distract students from their ongoing, productive inquiry and draw teachers’ attention away from students’ productive inquiry.	Medium
Publication 9	Kozaklı Ülger et al. (2026)	Türkiye	Teachers’ noticing and social interactions in the classroom: Insights from a skill-based science curriculum	Thinking Skills and Creativity	Exploratory case study	One novice and one expert fifth-grade science teacher	Written reports, teaching videos, and observation forms	three axis model	There is a strong correlation between students’ engagement in the lesson, occurrence of critical events, and teachers’ noticing.	High
Publication 10	Barnhart and van Es (2015)	United States	Studying teacher noticing: examining the relationship among preservice science teachers’ ability to attend, analyze and respond to student thinking	Teaching and Teacher Education	Comparative study using a teacher performance assessment	24 secondary science preservice teachers	Collection of preservice teachers’ PACT Teaching Event responses	Open coding, analytic memos, three-level coding framework, Mann–Whitney *U* test, Kendall’s Tau, and *t*-tests	The study showed that sophisticated analysis and response depended on sophisticated attention, but attention alone did not guarantee high-quality response.	High
Publication 11	Talanquer et al. (2013)	United States	Assessing students’ understanding of inquiry: what do prospective science teachers notice?	Journal of Research in Science Teaching	Exploratory qualitative study	43 prospective biology teachers in a secondary science teacher preparation program	Written responses to a video case and associated student-work artifacts	Constant comparison method; qualitative coding;	Prospective teachers focused more on inquiry process skills, especially designing investigations, and paid less attention to data analysis, conclusions, and the scientific quality of students’ ideas.	Medium
Publication 12	Luna et al. (2018)	United States	Teachers’ noticing of students’ thinking in science through classroom artifacts: in what ways are science and engineering practices evident?	Journal of Science Teacher Education	Qualitative study	20 elementary teachers	Semi structured interviews and collection of classroom artifacts from teachers’ garden-based science teaching	Qualitative coding	Teachers mainly noticed students’ thinking by focusing on what students were doing in artifacts. Planning and carrying out investigations appeared most often, while analyzing data and arguing from evidence were not represented.	High
Publication 13	Russ (2018)	United States	Characterizing teacher attention to student thinking: a role for epistemological messages	Journal of Research in Science Teaching	Qualitative theoretical-empirical study	13 high school science and mathematics teachers	Wearable video; interviews	Qualitative coding	Teacher attention to student thinking varied across the content, form, production, and relationships of students’ ideas, and these types of attention may send different epistemological messages.	High
Publication 14	Krist et al. (2023)	United States	Teacher noticing for supporting students’ epistemic agency in science sense making discussions	Journal of Science Teacher Education	Exploratory case study	Two experienced middle school science teachers	Point-of-view classroom video, stationary classroom video, and reflective interviews	Open coding, axial coding, teacher noticing framework, and descriptive analysis	attention to the epistemic nature of students’ responses and students’ epistemic stance or orientation may be especially important for supporting epistemic agency	High
Publication 15	Sharma (2025)	United States	Preservice science teachers’ notice and response to students’ scientific thinking within elementary science methods course	Research in Science Education	Qualitative content analysis with descriptive frequency counts	Preservice elementary teachers enrolled across five science methods course sections	36 sense making assignments;15 post-assessment analyses	Deductive coding using Kang and Anderson’s framework, followed by inductive coding and frequency counts	PSTs mainly noticed their own teaching, students’ behaviour, and whether students’ answers were correct. Their attention to students’ science ideas was limited, which often led to narrow interpretations and general responses	High
Publication 16	Park and Zhang (2025)	United States	Teacher noticing to scaffold knowledge-building inquiry in two grade 5 classrooms	Instructional Science	Qualitative case study	One veteran Grade 5 science teacher and 42 students in two Grade 5 classrooms	Teacher weekly reflection journals, classroom observations, video/audio recordings, and students’ online discourse in Knowledge Forum	Grounded theory approach	The teacher noticed students’ ideas, questions, participation, emotions, and needs, and used these observations to support deeper and broader student-driven inquiry.	Medium
Publication 17	Stammes and de Putter-Smits (2025)	Netherlands	Drawing meaning from student-generated drawings: exploring chemistry teachers’ noticing	Chemistry Education Research and Practice	Qualitative research	Three experienced chemistry teachers	Each teacher wore a head-mounted action camera and used a hand-held clicker to tag noteworthy classroom moments; lessons and subsequent interviews were video-recorded.	Transcription, multimodal data alignment, and deductive–inductive coding of drawing features and analytic stances.	Teachers identified chemistry-specific features in student drawings, mainly using evaluative rather than sense-making stances. Delayed noticing captured more features than in-the-moment noticing.	High

Written assignments, video cases, performance assessments, and reflections on teaching rehearsals primarily documented what teachers attended to, how they interpreted student thinking, and what responses they proposed. Barnhart and van Es (2015) found that “sophisticated analyses and responses to student ideas require high sophistication in attending to student ideas” and that “high sophistication in attending to student ideas does not guarantee more sophisticated analyses or responses” (p. 83). Benedict-Chambers (2016) similarly found that the tools used in post-rehearsal discussions “guided novices to collectively identify, interpret, and share insights to respond to critical issues of science teaching and learning” (p. 28). Arias et al. (2024) reported that preservice teachers were “more regularly privileging integrated sensemaking in rehearsal reflections compared to classroom enactment reflections” (p. 1).

Video-based protocols and analyses of student artifacts primarily documented teachers’ attention and interpretation, but generally did not trace these processes into subsequent instructional enactment (Luna et al., 2018; Mercier and Hinman, 2025; Mercier et al., 2026; Stammes and de Putter-Smits, 2025; Talanquer et al., 2013; see also Supplementary Table S1; see also Supplementary Table S1). By contrast, classroom-embedded studies provided more direct, although still varied, evidence of response. Krist et al. (2023) asked teachers to identify moments in which they were “making a decision about how to open up or close down space for students’ epistemic agency” and found that “teachers’ responses to students varied across pedagogical phenomena, and they also varied in their effectiveness” (p. 1). Park and Zhang (2025)reported that the teacher “kept weekly journals to record her reflective noticing of student inquiry and envisioning (planning) of possible ways to facilitate deeper knowledge building work” across an eight-month inquiry (p. 1). Their analysis traced pathways involving the interpretation of changes “within a temporal context” and the envisioning of “responsive actions that could be taken up with her students” (p. 1), and further sought to “trace how the moves were enacted with students” (p. 5). Kozaklı Ülger et al. (2026) collected data through teachers’ written reports, “their own teaching videos, self-reflection reports on their own lessons, and an observation form” (p. 1). They reported “a strong correlation between students’ engagement in the lesson, occurrence of critical events, and teachers’ noticing,” while limiting this conclusion to “the boundaries of these two cases and this specific context” (p. 1).

The evidentiary reach of each study therefore depended on its design. Attention and interpretation were documented most consistently; planned responses were common in teacher-education contexts; and enacted responses and subsequent student-level processes were examined less often. The literature consequently showed greater conceptual convergence than methodological consistency. These distinctions provide the basis for Research Question 2, which examines how the recognition, interpretation, and instructional use of student contributions may create opportunities for scientific thinking.

### 4.2 Research question 2: pathways through which teacher noticing supports students’ scientific thinking

Building on Section 4.1, the included studies suggest three related functions through which teacher noticing may create opportunities for students’ scientific thinking: recognizing scientific thinking in students’ classroom expressions, interpreting its disciplinary and epistemic significance, and using these interpretations to inform instructional responses. These functions may overlap and recur in classroom practice rather than unfold in a fixed order.

#### 4.2.1 Recognizing underlying scientific thinking in students’ classroom expressions

Students’ scientific thinking was not always expressed through canonical terminology or complete explanations. It could appear in everyday language, tentative explanations, and experiential examples (Mercier and Hinman, 2025; Mercier et al., 2026). It could also be represented through drawings, written work, digital media, and other classroom artifacts (Luna et al., 2018; Stammes and de Putter-Smits, 2025), or become visible through students’ participation in inquiry and scientific practices (Talanquer et al., 2013; Tang et al., 2010).

Students’ language provided one important source of evidence. Mercier and Hinman (2025) illustrated this through Ross, a preservice teacher who attended to the ways in which students communicated scientific thinking through “everyday language, talking like kids” (p. 959). He retained the intended meaning of a student’s explanation of friction despite the student’s use of “rags” rather than “drags”. However, Ross also described the student’s language as “unscientific” (p. 959). This case shows that a teacher may recognize the scientific meaning underlying a student’s expression while continuing to evaluate its wording against canonical scientific language.

Students’ experiences, representations, and classroom activities could also make their scientific thinking visible. Mercier et al. (2026) reported that one preservice teacher recognized an emerging understanding of condensation in a child’s everyday example: “The little boy used an example of his breath creating fog in the cold outside air. He definitely understands that there is a temperature connection to condensation even if he doesn’t use those words to say it” (p. 10). Luna et al. (2018) similarly concluded that “attending to what students are doing is one way teachers notice students’ thinking in science” (p. 148). Their findings suggested that classroom artifacts and teachers’ tendency to focus on what students were doing could be leveraged to support teachers in learning to notice students’ scientific thinking.

The reviewed evidence indicates that students’ underlying scientific thinking may become visible through what they say, represent, produce, and do in the classroom, rather than only through formally correct answers. Its visibility, however, is not automatic. It depends on the forms through which students express their thinking and on whether teachers treat those expressions as worthy of further attention. Recognition therefore establishes a possible object for interpretation, but does not by itself clarify what students understand or how they are constructing knowledge.

#### 4.2.2 Interpreting the disciplinary and epistemic meaning of students’ scientific thinking

Recognizing underlying scientific thinking in students’ classroom expressions does not by itself clarify what students understand or how they are constructing knowledge. Teachers must further interpret what students’ observations, explanations, representations, and participation reveal about scientific concepts and practices. Across the included studies, teachers interpreted several dimensions of student thinking that this review treats as manifestations of scientific thinking, including science content understanding, scientific practice use, inquiry reasoning, representational meaning, and epistemic participation (Benedict-Chambers and Aram, 2017; Krist et al., 2023; Sharma, 2025; Stammes and de Putter-Smits, 2025; Talanquer et al., 2013).

Teachers interpreted disciplinary meaning by examining what students’ observations and representations revealed about their subject-matter understanding. In a reflection on a peer-teaching rehearsal concerning water transport in celery, a preservice teacher proposed allowing students to share their observations because doing so could help him “assess their thinking and determine how to best build on their ideas” (Benedict-Chambers and Aram, 2017, p. 305). The observations were therefore interpreted not merely as records of what students had done, but as evidence that could inform the teacher’s understanding of their ideas and subsequent instructional planning. Stammes and de Putter-Smits (2025) provided a discipline-specific example from chemistry, in which experienced teachers attended to “chemistry-specific details” in student drawings (p. 504), such as visual forms, quantities, chemical entities represented at different length scales, and chemical properties and behaviours. These features provided a basis for interpreting how students understood and represented chemical entities and processes.

Teachers’ interpretations also extended beyond students’ conceptual understanding to the epistemic dimensions of their participation. Krist et al. (2023) found that teachers attended to the “disciplinary substance of students’ ideas,” the “epistemic nature of students’ ideas,” and students’ “epistemic stance or orientation during participation” (p. 15). The authors further suggested that attention to these epistemic dimensions may be particularly important for supporting students’ epistemic agency. This finding indicates that interpreting student thinking involves examining both what students understand and how they orient themselves toward classroom knowledge-building activity.

Collectively, the evidence suggests that interpretation involves determining both what students’ classroom expressions reveal about their scientific understanding and how students are constructing knowledge. This interpretive work provides a basis for instructional decision-making, but it does not by itself demonstrate that teachers acted on their interpretations. The next section therefore examines how teachers translated these interpretations into planned or enacted instructional responses.

#### 4.2.3 Translating interpretations into instructional responses

Teachers may use their recognition and interpretation of students’ scientific thinking to propose, plan, or enact instructional responses. In classroom-embedded studies, some responses also elicited further explanations, reorganized participation, or created additional opportunities for students to express their thinking. Instructional response was therefore not always an endpoint of noticing; it could also generate new evidence for subsequent recognition and interpretation. This third step therefore concerns whether and how teachers translated their understanding of student thinking into instructional responses. Across the included studies, these responses included retrospectively proposed revisions, planned teaching moves, immediate classroom actions, and longer-term adjustments to developing inquiry (Arias et al., 2024; Barnhart and van Es, 2015; Benedict-Chambers, 2016; Krist et al., 2023; Park and Zhang, 2025; Sharma, 2025). The evidence distinguished planned responses from responses documented in classroom practice.

Teacher-education studies primarily documented planned or retrospective responses. Barnhart and van Es (2015) found that “high sophistication in attending to student ideas does not guarantee more sophisticated analyses or responses” (p. 83). Their analysis nevertheless indicated that sophisticated responses depended on sophisticated analysis of student ideas. Arias et al. (2024) similarly found that preservice teachers were “more regularly privileging integrated sensemaking in rehearsal reflections compared to classroom enactment reflections” (p. 1). Together, these findings indicate that close attention and interpretation can support responsive planning, but do not guarantee that preservice teachers will formulate equally sophisticated responses, particularly when reflecting on the complexity of authentic classroom enactment.

Classroom-based studies provided more direct evidence of enacted responses. Krist et al. (2023) examined moments in which teachers decided how to “open up or close down space for students’ epistemic agency” (p. 1). Teachers’ responses varied across classroom situations and also differed in their effectiveness, indicating that responding to noticed student thinking did not automatically support students’ participation in sensemaking. Park and Zhang (2025) provided longitudinal evidence from an eight-month knowledge-building inquiry in two Grade 5 classrooms. Drawing on the teacher’s weekly reflection journals, the study traced how a veteran teacher attended to students’ evolving ideas and questions, participation, needs and emotions, and the development of collective inquiry. The authors described these pathways as involving the interpretation of changes “within a temporal context” and the envisioning of “responsive actions that could be taken up with her students” (p. 1). Classroom observations, video and audio recordings, and students’ online discourse were further used to trace how selected planned moves were enacted with students.

Across these studies, teachers used their interpretations of student thinking to propose, plan, or enact instructional responses. Such responses could open or constrain opportunities for students to develop ideas, engage in sensemaking, and participate in individual or collective knowledge construction. However, a key tension emerged across studies: teachers’ attention to students’ thinking did not necessarily lead to effective instructional responses (Barnhart and van Es, 2015; Krist et al., 2023).

Planned responses were documented more frequently than enacted responses, and direct evidence of sustained changes in students’ scientific thinking remained limited. Recognition, interpretation, and instructional response can therefore be distinguished as three related steps, but their relationship appears recursive rather than fixed. Instructional responses may alter what students express and what teachers can subsequently notice. The synthesis identifies recurring patterns in how teacher noticing is conceptualized and enacted, while also revealing important limits in the available evidence. The following discussion interprets these patterns in relation to broader theories of teacher noticing, scientific thinking, and responsive science instruction.

## 5 Discussion

Regarding the first research question, the findings position science teacher noticing as a knowledge-guided, discipline-specific, socially situated, and developable professional practice. It is not a neutral act of observing classroom events. Consistent with Chan et al. (2021), the literature converged around attention, interpretation, and instructional response. However, studies differed in how they defined these components and represented them empirically. König et al. (2022) explain this variation through cognitive-psychological, sociocultural, discipline-specific, and expertise-related perspectives. Kozaklı Ülger et al. (2026) further show that teachers’ goals, schemas, and professional knowledge guide information selection, monitoring, organization, and interpretation. In science classrooms, this process depends on content knowledge, pedagogical content knowledge, knowledge of students, and understanding of scientific practices (Chan et al., 2021; König et al., 2022). Mendez and van Es (2024) add that teachers’ personal histories, understandings of disciplinary narratives, and sociopolitical contexts shape what becomes visible and how students’ ideas are interpreted. Science teacher noticing is therefore cognitive in operation, disciplinary in substance, sociocultural and relational in orientation, and developable through professional learning.

Regarding the second research question, this review responds to Chan et al. (2021) call to examine teachers’ attention to students’ scientific thinking and reasoning. As conceptualized in Section 2, scientific thinking extends beyond canonical terminology and correct answers. Recognition concerns whether teachers identify scientific meaning in students’ questions, explanations, drawings, models, uncertainty, and inquiry participation. Interpretation concerns what these expressions reveal about disciplinary understanding, reasoning, and epistemic participation, whereas instructional response concerns how teachers use their interpretations to guide interaction and create opportunities for further sensemaking. These functions are connected, but they do not form a fixed sequence. Professional learning may strengthen teachers’ attention and interpretation (Luna and Sherin, 2017), but sophisticated attention does not guarantee equally sophisticated analysis or response (Barnhart and van Es, 2015). Classroom-based studies further indicate that instructional moves may expand or constrain students’ epistemic agency and that attention, interpretation, and responsive planning may recur across extended inquiry (Krist et al., 2023; Park and Zhang, 2025).

van Es and Sherin (2025) concept of shaping deepens this recursive account by showing how teachers may elicit details, reorganize participation, and use material or social resources to gain further access to student thinking. Kozaklı Ülger et al. (2026) provide complementary science-specific evidence for this interactional view: interactive and dialogic communication accompanied more visible critical events and more elaborated student responses. Although these relationships remain descriptive, they illustrate how discourse may influence what student thinking becomes available to notice. Teacher noticing may therefore shape which questions are pursued, which explanations are valued, which evidence is requested, which ideas are extended, and who receives opportunities to speak, revise, challenge, and contribute to collective knowledge construction. These actions generate new student expressions that may become objects for further recognition and interpretation. Teacher noticing also involves affective dimensions of classroom inquiry. Park and Zhang (2025) documented how a teacher attended to students’ emotions, frustration, and participation needs, and used these observations to interpret students’ experiences and plan responsive support for collaborative inquiry. The process can be understood as recursive: teachers’ knowledge and sociocultural lenses may influence noticing, while noticing may shape the conditions through which scientific thinking becomes visible. The evidence does not establish a direct causal mechanism linking teacher noticing to sustained student development; however, it supports the cautious conclusion that noticing may shape the epistemic, interactional, and relational conditions through which scientific thinking becomes visible and may develop.

This recursive account also has methodological implications. Research materials and protocols do not provide neutral or equivalent evidence of teacher noticing. Short clips, full classroom recordings, student artifacts, rehearsals, and written reflections make different aspects of noticing visible. Open prompts, focused prompts, and selected critical events also direct attention differently. van Es and Amador (2025) argue that an event becomes critical in relation to the observer, purpose, and mode of examination. van Es and Sherin (2025) similarly note that the initial selection of video clips is itself an act of noticing. These choices help explain why attention and interpretation were documented more consistently than enacted responses or sustained student-level change. Evidence from video tasks or written reflections should therefore not be treated as equivalent to evidence from classroom enactment. Longitudinal and classroom-embedded designs are needed to examine how noticing unfolds within ongoing interaction.

The reviewed evidence also has methodological and contextual limitations. Most of the included studies used qualitative designs, and many were conducted in the United States. Furthermore, several involved preservice teachers. While these studies provide valuable, context-specific insights, further research is needed to determine the applicability of the proposed recursive account across different educational systems, grade levels, science disciplines, and in-service teaching contexts.

The principal contribution of this review is a student-centered and recursive account of science teacher noticing. It connects teachers’ professional resources with the recognition, interpretation, and shaping of students’ scientific thinking. For teacher education, the implication is not simply that teachers should notice more student ideas. Professional learning should strengthen the knowledge needed to recognize the scientific significance of students’ ideas. It should also develop evidence-based, asset-oriented interpretation and the interactional capacity to elicit and extend student reasoning. Video analysis, student-artifact analysis, rehearsal, classroom enactment, and reflection may therefore be most useful when organized as an iterative learning cycle.

## 6 Conclusion

This integrative narrative review synthesized 17 empirical studies examining how teacher noticing has been conceptualized and operationalized in K–12 science education and how it may shape opportunities for students’ scientific thinking. The reviewed literature supports a recursive account in which recognition, interpretation, and instructional response are analytically distinguishable but interconnected: teachers’ responses may influence what students express and, consequently, what becomes available for subsequent noticing. The evidence also suggests that noticing is discipline-specific and shaped by teachers’ professional knowledge, contextual expectations, and sociocultural lenses. However, most studies documented teachers’ attention, interpretation, or proposed responses rather than sustained changes in student-level outcomes. The review therefore identifies consistent patterns across the included studies, enabling conditions, and plausible instructional pathways rather than establishing direct causal effects on the development of scientific thinking. Future research should examine how teachers notice and respond to students’ emotions, social interactions, and ethical considerations, particularly in socioscientific issues (SSI) discussions.
